# Maternal smoking during pregnancy and offspring risk of intellectual disability: a UK-based cohort study

**DOI:** 10.3389/fpsyt.2024.1352077

**Published:** 2024-06-25

**Authors:** Paul Madley-Dowd, Richard Thomas, Andy Boyd, Stanley Zammit, Jon Heron, Dheeraj Rai

**Affiliations:** ^1^ Centre for Academic Mental Health, Population Health Sciences, Bristol Medical School, University of Bristol, Bristol, United Kingdom; ^2^ National Institute for Health and Care Research (NIHR) Bristol Biomedical Research Centre, University Hospitals Bristol and Weston National Health Service (NHS) Foundation Trust and University of Bristol, Bristol, United Kingdom; ^3^ Medical Research Council Integrative Epidemiology Unit at the University of Bristol, Bristol, United Kingdom; ^4^ UK Longitudinal Linkage Collaboration, Population Health Sciences, Bristol Medical School, University of Bristol, Bristol, United Kingdom; ^5^ Medical Research Council (MRC) Centre for Neuropsychiatric Genetics and Genomics, Cardiff University, Cardiff, United Kingdom; ^6^ Avon and Wiltshire Partnership NHS Mental Health Trust, Bath, United Kingdom

**Keywords:** ALSPAC, intellectual disability, negative control, prenatal exposure, smoking

## Abstract

**Background:**

Observational studies have described associations of maternal smoking during pregnancy with intellectual disability (ID) in the exposed offspring. Whether these results reflect a causal effect or unmeasured confounding is still unclear.

**Methods:**

Using a UK-based prospectively collected birth cohort (the Avon Longitudinal Study of Parents and Children) of 13,479 children born between 1991 and 1992, we assessed the relationship between maternal smoking at 18 weeks’ gestation and offspring risk of ID, ascertained through multiple sources of linked information including primary care diagnoses and education records. Using confounder-adjusted logistic regression, we performed observational analyses and a negative control analysis that compared maternal with partner smoking in pregnancy under the assumption that if a causal effect were to exist, maternal effect estimates would be of greater magnitude than estimates for partner smoking if the two exposures suffer from comparable biases.

**Results:**

In observational analysis, we found an adjusted odds ratio for ID of 0.75 (95% CI = 0.49–1.13) for any maternal smoking and 0.97 (95% CI = 0.71–1.33) per 10-cigarette increase in number of cigarettes smoked per day. In negative control analysis, comparable effect estimates were found for any partner smoking (OR = 0.94; 95% CI = 0.63–1.40) and number of cigarettes smoked per day (OR = 0.94; 95% CI = 0.74–1.20).

**Conclusions:**

The results are not consistent with a causal effect of maternal smoking during pregnancy on offspring ID.

## Background

Maternal smoking in pregnancy is reported in over 8% of pregnancies in Europe (1). It has a well-established causal relationship with low birthweight (2, 3) and a more tentative association with other adverse pregnancy and offspring health outcomes such as pregnancy complications (4) and sudden infant death syndrome (5). Establishing which offspring health outcomes are caused by maternal smoking in pregnancy may (i) provide insight as to which adverse health outcomes may be reduced through smoking cessation initiatives, (ii) aid in understanding the mechanisms by which these conditions occur, and (iii) create the opportunity for mothers to have an informed choice about the potential consequences of deciding to or not to give up smoking during pregnancy.

An offspring outcome with under-researched aetiology is intellectual disability (ID). ID is a developmental condition defined as having an arrested or incomplete development of the mind alongside functional impairment in facets that contribute to overall intelligence such as cognition, language, and social ability (6). ID manifests during the developmental period and is not the result of later changes to the brain as a result of injury or disease. Further details on ID issues surrounding its definition have been discussed elsewhere (7).

An association of increased risk of ID in the offspring of mothers who smoked during pregnancy has been suggested in the literature (8–15). A systematic review has suggested that smoking during pregnancy is associated with a small increase in the risk of offspring ID (12), although the studies included (8–11) did not adequately account for confounding or information bias. Two better-quality studies not included in the review found an association between smoking in pregnancy and offspring risk of ID, but each suggested that this may be the result of residual confounding (13, 14). We have recently published two further studies investigating the association between maternal smoking in pregnancy and risk of offspring ID using nationally representative Danish and Swedish registry data (16, 17). These studies employed exposure-discordant sibling designs to account for genetic and environmental confounding shared between siblings. The results of these studies suggested that, whereas increased odds of ID were found among offspring of mothers who smoked during pregnancy in conventional analyses, the sibling analyses suggested that these associations were attributable to characteristics that differed between families as opposed to individual-level exposure to smoking in pregnancy—suggesting that the association did not reflect a causal effect.

Triangulation of evidence from different methods, each with their own biases, can help to establish whether associations reflect causal effects (18–20). This is particularly important when randomized control experiments are not ethically plausible. In the present study, we aimed to use the negative control design in a UK-based pregnancy cohort, the Avon Longitudinal Study of Parents and Children (ALSPAC), to provide further evidence as to whether associations between maternal smoking in pregnancy and offspring intellectual disability reflect causal effects. The negative control design describes analyses that compare the magnitude of an estimate of an exposure–outcome association against the estimate of another association in which the exposure has been replaced with a variable such that the new association is not plausibly causal via the hypothesized mechanism (21, 22). We end by summarizing evidence across causal inference methods to triangulate evidence.

## Methods

### Cohort

The ALSPAC cohort (23–25) recruited 14,541 pregnant women resident in and around the City of Bristol, South West UK, with expected dates of delivery 01/04/1991 to 31/12/1992. There were 14,203 unique mothers initially enrolled in the study. Mothers invited partners to complete questionnaires at the start of the study and 12,113 partners have provided data to the study. Please note that the ALSPAC study website contains details of all the data that are available through a fully searchable data dictionary (http://www.bristol.ac.uk/alspac/researchers/our-data/).

**Figure 1:**
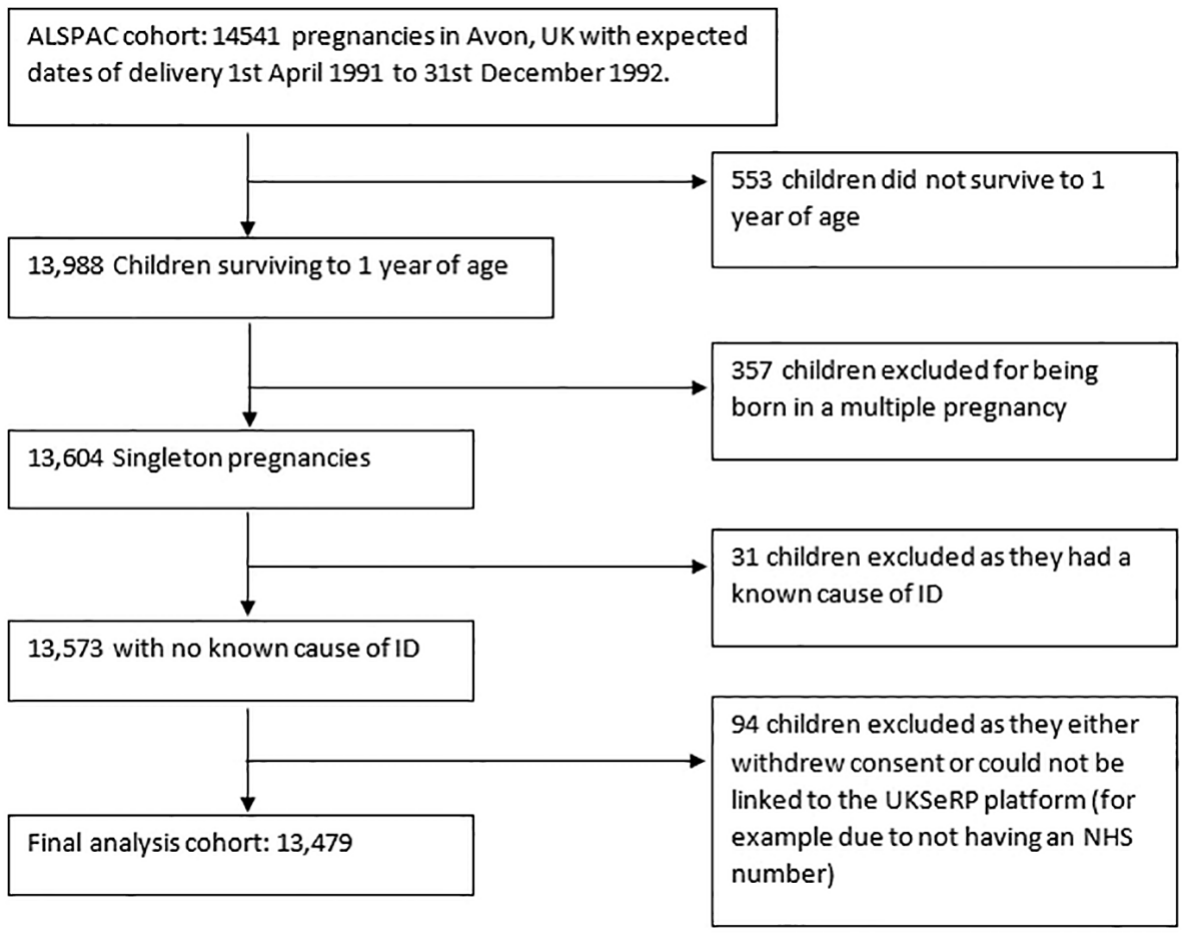
Flow chart of exclusions.

The unit of analysis for this investigation is the index offspring of the pregnancies. Eligibility criteria for children in this investigation were (1) surviving to 1 year of age, (2) being a singleton pregnancy, (3) not having a known cause of ID (see (7) for derivation of known causes of ID), (4) not having withdrawn consent by the time of analysis, and (5) having an NHS number so that ALSPAC data could be linked to outcome information on the UK Secure eResearch Platform. Children with a known cause of ID were excluded, as this is a group in which ID is likely regardless of exposure to maternal smoking during pregnancy. This left a total sample size of 13,479 children (see **Figure 1** for a flowchart of exclusions).

### Exposure definition—maternal and partner smoking during pregnancy

Binary (yes/no) and count (number of cigarettes per day) variables for maternal and partner smoking during pregnancy were derived from questionnaire responses intended to be complete at 18 weeks’ gestation (actual gestation at completion varied). Detailed description of the questionnaires, derivation process, and time of completion is provided in the **Supplementary Methods**. We use the term partner as opposed to paternal throughout to acknowledge that in ALSPAC, the mother’s partner may not be the biological father of the child.

### Outcome definition—offspring intellectual disability

The derivation of a multiple-sourced variable for ID has been described in detail elsewhere (7). Briefly, data linkage was employed to combine information on IQ scores assessed by ALSPAC fieldworkers when the children were age 8 and 15, diagnoses of ID using Read (26, 27) and ICD (6) codes in general practitioner (GP) and hospital episode statistic (HES) records, statements of special educational needs for cognitive and learning needs (28) from school census records, and free text information recorded in questionnaires by participants and their guardians (including mothers, partners, and other primary carers) across the lifetime of the study. A child was indicated as having ID if two or more of the sources indicated having ID. Known causes of ID used in the eligibility criteria (including genetic, metabolic, or chromosomal abnormalities associated with ID) were also identified from GP, HES, and free text information.

### Covariate variable definitions

The variables used as covariates in models were the following: child sex assigned at birth, maternal age at the time of birth, maternal parity, maternal depressive symptoms at 18 weeks’ gestation, maternal alcohol use recorded at 18 weeks’ gestation, maternal reported financial difficulties recorded at 32 weeks’ gestation, maternal education recorded at 32 weeks’ gestation, and maternal occupational class recorded at 32 weeks’ gestation. Detailed description of variable derivations can be found in the **Supplementary Methods**.

### Statistical analysis

All analyses were performed using R version 3.5.3 (29).

#### Observational analyses

Logistic regression models of ID on exposure were repeated for the binary measure of smoking in pregnancy and the number of cigarettes smoked per day. Models were performed using four adjustment strategies: (i) unadjusted, (ii) adjusted for maternal characteristics (maternal age at birth, parity, maternal depressive symptoms, maternal alcohol use during pregnancy and child sex), (iii) adjusted for socioeconomic factors (financial difficulties, education, and occupational class), and (iv) adjusted for both maternal characteristics and socioeconomic factors.

#### Negative control analyses

Logistic regression models of ID on maternal and partner smoking during pregnancy, mutually adjusted for each other to reduce bias from assortative mating (22), were fitted using the same four adjustment strategies as for the observational analyses. The models were repeated for the binary and count forms of the exposure variable. In these models, a causal effect is implied for maternal smoking in pregnancy if a substantially higher effect for maternal smoking than partner smoking is found as it is assumed that partner smoking has either no, or a much smaller, *in utero* effect than maternal smoking. We used the pair sexual isolation index (I_PSI_) to assess the strength of assortative mating for smoking behavior (30, 31).

### Missing data assessment and multiple imputation analyses

To assess the likelihood of bias from missing data, we compared the prevalence/means of exposure, outcome, confounders, and auxiliary variables between those included in the sample and those excluded. We further performed logistic regression of being included in complete record analysis on each variable without adjustment. Complete record analysis has been shown to be biased when the probability of missing data is jointly dependent on both the exposure and the outcome for logistic regression (32).

All observational analyses and negative control analyses were conducted as complete records analyses and repeated using multiple imputation. Multiple imputation was implemented to reduce bias and improve efficiency (33). Previous work has shown that, provided the data meet the missing at random (MAR) assumption, multiple imputation can produce unbiased results even at large proportions of missing data (34). Data were imputed using fully conditional specification (35, 36) carried out using the R package “mice” (37) with 100 imputations. The exposure, outcome, and all maternal and partner covariates were included in the imputation model to maintain consistency between the imputation model and the most complex analysis model (the fully adjusted negative control model). Each variable was included as a predictor of all other variables.

Auxiliary variables were also included in the imputation model in order to improve the plausibility of the MAR assumption (36). We included two auxiliary variables for socioeconomic status as the analysis model variables for this (financial difficulties, education, and occupation) were often missing. The auxiliary variables were home ownership status and present maternal marital status, both recorded at approximately 6 weeks’ gestation. Further details of the multiple imputation procedure and auxiliary variables are detailed in the **Supplementary Methods**.

We report the fraction of missing information (FMI) for the exposure coefficient. The FMI is a parameter-specific measure that quantifies the loss of information due to missing data while accounting for information recovered by multiple imputation (38, 39). Values of FMI range between 0 and 1 with values close to 1 indicating that observed data in the imputation model does not provide much information about the missing data.

## Results

Of the 13,479 included children, 137 (1.0%) had an intellectual disability, of which 36 (26.3%) were exposed to maternal smoking during pregnancy and 53 (38.7%) were exposed to partner smoking during pregnancy. There were 373 (2.8%) children considered to have missing data for the outcome due to insufficient information on ID being available.

Descriptives of the cohort separated by maternal and partner smoking status during pregnancy are presented in **Table 1**. The table shows that 25.2% of children were exposed to maternal smoking during pregnancy, 68.9% were not exposed, and 5.8% had no exposure data available. Maternal smokers were more likely to be younger, have prenatal depression symptoms, have used alcohol during pregnancy, have a lower level of education, have a manual occupation, and to have experienced financial difficulties during pregnancy.

**Table 1:** Descriptive statistics separated by exposure status (maternal/partner smoking at 18 weeks’ gestation).

Characteristic	Maternal non-smoker	Maternal smoker	Missing maternal smoking data	Partner non-smoker	Partner smoker	Missing partner smoking data
	N = 9,293	N = 3,401	N = 785	N = 7,450	N = 4,910	N = 1,119
**Number of times smoked per day, median (IQR)**	–	5 (0–10)	–	–	10 (5–20)	–
Parental age, N (%)						
**<25**	1,608 (17.3)	1,319 (38.78)	344 (43.82)	324 (4.35)	440 (8.96)	40 (3.57)
**25–39**	3,741 (40.26)	1,201 (35.31)	262 (33.38)	1,556 (20.89)	911 (18.55)	56 (5.00)
**30–34**	2,893 (31.13)	647 (19.02)	126 (16.05)	1,786 (23.97)	760 (15.48)	39 (3.49)
**≥35**	1,051 (11.31)	234 (6.88)	53 (6.75)	1,113 (14.94)	558 (11.36)	24 (2.14)
**Missing**	0 (0)	0 (0)	0 (0)	2,671 (35.85)	2,241 (45.64)	960 (85.79)
Parental highest education, N(%)						
**Vocational**	772 (8.31)	376 (11.06)	37 (4.71)	418 (5.61)	364 (7.41)	≤5
**CSE/O level**	4,381 (47.14)	1,986 (58.39)	190 (24.2)	2,233 (29.97)	1,943 (39.57)	24 (2.14)
**A level/degree**	3,576 (38.48)	605 (17.79)	55 (7.01)	3,228 (43.33)	1,232 (25.09)	≤5
**Missing**	564 (6.07)	434 (12.76)	503 (64.08)	1,571 (21.09)	1,371 (27.92)	1,094 (97.77)
Occupation, N (%)						
**Non-manual**	6,165 (66.34)	1,505 (44.25)	101 (12.87)	4,151 (55.72)	1,649 (33.58)	91 (8.13)
**Manual**	1,200 (12.91)	685 (20.14)	49 (6.24)	2,363 (31.72)	2,151 (43.81)	151 (13.49)
**Missing**	1,928 (20.75)	1,211 (35.61)	635 (80.89)	936 (12.56)	1,110 (22.61)	877 (78.37)
Financial difficulties, N (%)						
**No**	7,901 (85.02)	2,396 (70.45)	192 (24.46)	6,419 (86.16)	3,675 (74.85)	395 (35.3)
**Yes**	628 (6.76)	496 (14.58)	44 (5.61)	465 (6.24)	597 (12.16)	106 (9.47)
**Missing**	764 (8.22)	509 (14.97)	549 (69.94)	566 (7.6)	638 (12.99)	618 (55.23)
Depression, N (%)						
**No**	7,679 (82.63)	2,415 (71.01)	0 (0)	5,725 (76.85)	3,404 (69.33)	≤5
**Yes**	933 (10.04)	684 (20.11)	0 (0)	174 (2.34)	208 (4.24)	≤5
**Missing**	681 (7.33)	302 (8.88)	785 (100)	1,551 (20.82)	1,298 (26.44)	1,116 (99.73)
Alcohol use in pregnancy, N (%)						
**No**	4,362 (46.94)	1,331 (39.14)	0 (0)	275 (3.69)	181 (3.69)	≤5
**Yes**	4,824 (51.91)	2,032 (59.75)	0 (0)	5,541 (74.38)	3,362 (68.47)	≤5
**Missing**	107 (1.15)	38 (1.12)	785 (100)	1,634 (21.93)	1,367 (27.84)	1,116 (99.73)
Ethnicity, N(%)						
**White**	8,429 (90.7)	2,897 (85.18)	255 (32.48)	5,676 (76.19)	3,441 (70.08)	≤5
**All other ethnic groups combined**	237 (2.55)	58 (1.71)	15 (1.91)	160 (2.15)	111 (2.26)	≤5
**Missing**	627 (6.75)	446 (13.11)	515 (65.61)	1,614 (21.66)	1,358 (27.66)	1,116 (99.73)
Parity, N(%)						
**0**	4,118 (44.31)	1,490 (43.81)	0 (0)	3,304 (44.35)	2,100 (42.77)	204 (18.23)
**1**	3,314 (35.66)	1,028 (30.23)	0 (0)	2,658 (35.68)	1,614 (32.87)	70 (6.26)
**≥2**	1,738 (18.7)	785 (23.08)	0 (0)	1,376 (18.47)	1,086 (22.12)	61 (5.45)
**Missing**	123 (1.32)	98 (2.88)	785 (100)	112 (1.5)	110 (2.24)	784 (70.06)
Child sex, N(%)						
**Female**	4,573 (49.21)	1,592 (46.81)	366 (46.62)	3,636 (48.81)	2,372 (48.31)	523 (46.74)
**Male**	4,720 (50.79)	1,809 (53.19)	419 (53.38)	3,814 (51.19)	2,538 (51.69)	596 (53.26)

Note that values labelled as ≤5 may include 0.

Child prenatal exposure to partner smoking was more common than to maternal smoking (36.4% vs. 25.2%) but was more often missing. Partners tended to smoke more cigarettes per day than mothers if they did smoke [median number smoked (inter quartile range): 10 (5–20) vs. 5 (0–10)]. The overall pattern of confounder distributions between smokers and non-smokers was similar between mothers and partners for most characteristics; smokers tended to be younger, have depression during pregnancy, have a lower education, have a manual occupation, and have experienced financial difficulties. The actual distributions were not similar, however, as partners tended to be older than mothers, were more likely to have post age 16 formal education to A-level or degree standard, and work a manual job. It is unclear whether this disparity is due to actual differences in the distributions or due to substantially lower responses from partners than mothers. Partners were less likely to respond to questions on smoking, alcohol consumption and depression. The number reporting depression was lower for partners than mothers whereas alcohol use was more common among partners. A full description of the missing data assessment is presented in the **Supplementary Results** and in **Supplementary Tables S1**, **S2**.

A cross tabulation of maternal and partner smoking is presented in **Supplementary Table S3**, which shows that there is evidence for positive assortative mating between parents for smoking behavior (mother and partner are likely to exhibit similar smoking behaviors; I_PSI_ = 0.41), justifying our use of mutual adjustment in negative control analyses.

### Observational analyses

The results of the observational analyses for both binary and count exposure are presented in **Table 2**. Both complete records analysis and multiple imputation analysis found no association between the binary measure of maternal smoking during pregnancy and offspring odds of ID. Models that were unadjusted and adjusted for confounders were all consistent with no effect (OR for fully adjusted model = 0.83; 95% CI = 0.48–1.44). Results for the count exposure showed a 1.29-fold increased odds of ID per 10 cigarettes smoked per day in pregnancy (95%CI = 0.82–2.01) in the unadjusted model. This association was attenuated somewhat after adjusting for maternal characteristics and attenuated substantially towards the null following adjustment for socioeconomic characteristics (OR for the fully adjusted model = 1.01; 95%CI = 0.63–1.60). Results from multiple imputation analyses were consistent with those from complete records analysis. Values of FMI were close to 0, indicating that we did not lose substantial information on the exposure-outcome association to missing data.

**Table 2:** Results of the observational analyses of maternal smoking during pregnancy and offspring intellectual disability.

Model	Complete records analysis	Multiple imputation analysis	
	OR (95% CI)	OR (95% CI)	FMI
Binary exposure			
Unadjusted	1.07 (0.64–1.79)	1.05 (0.72–1.55)	0.084
Adjusted for maternal characteristics	1.05 (0.61–1.80)	0.95 (0.64–1.42)	0.088
Adjusted for socioeconomic characteristics	0.78 (0.45–1.32)	0.73 (0.49–1.09)	0.109
Adjusted for all confounders	0.83 (0.48–1.44)	0.75 (0.49–1.13)	0.102
Count exposure (10 cigarettes per day)			
Unadjusted	1.29 (0.82–2.01)	1.30 (0.97–1.73)	0.086
Adjusted for maternal characteristics	1.22 (0.78–1.91)	1.16 (0.86–1.57)	0.085
Adjusted for socioeconomic characteristics	0.99 (0.62–1.58)	0.99 (0.72–1.36)	0.113
Adjusted for all confounders	1.01 (0.63–1.60)	0.97 (0.71–1.33)	0.104

Complete records analysis with binary exposure—N = 8808.

Complete records analysis with count exposure—N = 8785.

Multiple imputation analysis N = 13,479.

OR, odds ratio; CI, confidence interval; FMI, fraction of missing information for the exposure coefficient.

For the count exposure, the odds ratio reflects the change in odds per 10 cigarette increase in number of cigarettes smoked per day.

### Negative control analyses

**Table 3** shows that in complete records analyses, the effect estimates for the binary exposure suggested that maternal smoking was associated with reduced odds of ID in offspring (fully adjusted OR = 0.69; 95% CI = 0.26–1.82) whereas partner smoking was associated with increased odds (fully adjusted OR = 1.18; 95% CI = 0.54–2.56). It is important to note that both effect estimates are consistent with the null and consistent with each other, providing evidence against a causal effect of smoking in pregnancy on offspring risk of ID. Multiple imputation analyses that account for missing data brought both maternal and partner effect estimates closer to the null and closer to each other.

**Table 3:** Results of the negative control analyses of maternal smoking during pregnancy and offspring intellectual disability.

Model	Complete records analysis		Multiple imputation analysis	
	Maternal OR (95% CI)	Partner OR (95% CI)	Maternal OR (95% CI)	Partner OR (95% CI)
Binary exposure				
Unadjusted	0.81 (0.31–2.08)	1.39 (0.66–2.91)	0.99 (0.66–1.50)	1.15 (0.79–1.69)
Adjusted for maternal characteristics	0.74 (0.28–1.97)	1.37 (0.64–2.92)	0.90 (0.59–1.38)	1.13 (0.76–1.67)
Adjusted for socioeconomic characteristics	0.69 (0.27–1.80)	1.17 (0.55–2.50)	0.73 (0.48–1.12)	0.91 (0.62–1.34)
Fully adjusted for confounders	0.69 (0.26–1.82)	1.18 (0.54–2.56)	0.74 (0.48–1.14)	0.94 (0.63–1.40)
Count exposure (10 cigarettes per day)				
Unadjusted	1.20 (0.54–2.66)	1.40 (0.93–2.10)	1.24 (0.90–1.71)	1.08 (0.85–1.36)
Adjusted for maternal characteristics	1.12 (0.49–2.56)	1.38 (0.90–2.11)	1.11 (0.80–1.54)	1.06 (0.83–1.35)
Adjusted for socioeconomic characteristics	1.09 (0.48–2.45)	1.28 (0.83–1.96)	1.01 (0.72–1.40)	0.93 (0.73–1.19)
Fully adjusted for confounders	1.09 (0.47–2.52)	1.27 (0.81–1.97)	0.97 (0.70–1.35)	0.94 (0.74–1.20)

All models are mutually adjusted for maternal and partner exposure.

Complete records analysis with binary exposure—N = 5,151.

Complete records analysis with count exposure—N = 5,064.

Multiple imputation analysis N = 13,479.

OR, odds ratio; CI, confidence interval.

For the count exposure the odds ratio reflects the change in odds per 10-cigarette increase in number of cigarettes smoked per day.

Results for the count exposure show a greater OR per 10 cigarettes smoked per day for partner smoking than maternal smoking (unadjusted maternal OR and 95% CI = 1.20, 0.54–2.66; unadjusted partner OR and 95% CI = 1.40, 0.93–2.10). These estimates were attenuated towards the null following adjustment for confounders (fully adjusted maternal OR and 95% CI = 1.09, 0.47–2.52; fully adjusted partner OR and 95% CI = 1.27, 0.81–1.97). As with the binary exposure, these estimates are consistent with the null and with each other providing evidence against a causal effect of maternal smoking in pregnancy. Multiple imputation analyses again brought the estimates closer to the null and closer together.

## Discussion

In this cohort study, our results did not provide evidence for an association between maternal smoking during pregnancy and offspring intellectual disability. By using a negative control design that compared maternal effects with partner effects, which were assumed to be smaller in magnitude if a causal effect were to exist, we have explored an association reported in previous studies that may be afflicted by unmeasured or residual confounding. Results of the negative control design were also not consistent with a causal effect.

### Comparison with previous literature

Prior work has suggested an increased risk of ID following prenatal exposure to maternal smoking during pregnancy. A meta-analysis (12) of two case–control studies (8, 9) and two prospective birth cohort studies (10, 11) suggested a small increased risk (OR = 1.10 95% CI 1.06–1.15), although the included studies did not adequately account for confounding and may also suffer from selection and recall bias. The estimate from our unadjusted model using a binary exposure is close to the meta-analyzed value. Adjusted OR estimates of studies not included in the meta-analysis range from 1.27 (95% CI = 1.19–1.34) (14) to 1.35 (95% CI = 1.28–1.42) (16). These effect estimates are greater in magnitude than the effects estimated in the current study but do overlap with the confidence intervals we have produced owing in part to our smaller sample size and larger standard errors.

The results of the present study and the suggestion of no causal effect by our negative control analyses are consistent with other studies using causal inference methods (16, 17), namely, the exposure discordant sibling design, which accounts for unmeasured genetic and environmental confounding shared between siblings. The result of the present study therefore provides further evidence to suggest that observational associations between maternal smoking in pregnancy and offspring risk of ID reflect unmeasured or residual confounding. Each of these studies may be susceptible to their own biases; for example, sibling designs are susceptible to bias from non-shared confounding between siblings and carryover effects where the outcome or exposure of one pregnancy influences the exposure status in following pregnancies, although in both cases bias is more likely to be away from the null. The negative control assumes that bias from all sources (confounding, selection, and measurement error) are equivalent for the maternal and partner exposure. This assumption may not hold perfectly in our study as there were differing relationships between the maternal and partner exposure variables and missing data. In spite of this, both maternal and partner effect estimates were close to the null following adjustment for confounding suggesting a lack of association or causal effect of smoking in pregnancy on offspring risk of ID.

### Strengths and limitations

We have used a pregnancy cohort with prospectively collected data which will reduce the chance of differential measurement error in the exposure (recall bias). The ALSPAC cohort contains information on an extensive range of maternal and partner characteristics, which has enabled us to adjust for a wide set of confounding variables which were also prospectively collected. Despite this, we likely did not fully account for confounding in our adjustment set, in part due to the difficult nature of characterizing socioeconomic position, and therefore, residual and unmeasured confounding is possible. To overcome this, we implemented a causal inference technique, the negative control design, to try to account for bias from unmeasured confounding, selection, and measurement error. We acknowledge that these biasing structures for maternal and partner smoking may not overlap perfectly. The negative control design accounts for some level of genetic confounding as both mother and father contribute 50% of their genetics to the genetics of the child; comparing maternal with paternal smoking effects means that each effect should be confounded by genetics to a similar extent. However, not all partners in this study may have been the biological father of the child and so the partner smoking effect may suffer from less genetic confounding than the maternal smoking effect. It is also possible that mothers in the study underreported how much they smoked during pregnancy to a greater extent than partners due to differences in social pressures and desirability (40). This could result in a greater bias towards the null for the maternal effect than the partner effect.

The ALSPAC cohort has a relatively small sample size compared with other studies using national registers. This means that we have more uncertainty in our estimates than some prior studies. It is important in the context of a lack of statistical power to remember that absence of evidence is not equivalent to evidence of absence; however, given that these results agree with our prior findings for a lack of causal effect, we are encouraged in our conclusions. Furthermore, ALSPAC overrepresents mothers with White ethnicity than the UK population as a whole, due in large part to the demographics of the eligible population in the catchment area at the time, and so may be less generalizable than studies using national registers.

ALSPAC is also afflicted by socioeconomic and health patterning in attrition (41). Our study has been strengthened by our use of data linkage to healthcare and education records to reduce the quantity of missing data in the outcome. This will have improved the statistical efficiency of our estimates (thereby reducing uncertainty) and likely reduced bias in analyses by reducing the dependency of the probability of missing data in the outcome on the underlying value of the outcome itself (32). We further accounted for missing data in the exposure and confounders variables using multiple imputation, improving statistical efficiency. We found close estimates between complete records analysis and MI, but it is important to note that this does not provide evidence that estimates are unbiased by missing data as both complete records and multiple imputation analysis could be biased to a similar extent if the MAR assumption was not met. We used auxiliary variables to improve the plausibility that data in exposure and confounding variables was MAR.

## Conclusions

The results of this study provide further evidence that the association between maternal smoking during pregnancy and offspring risk of ID is unlikely to reflect a causal effect. This finding does not imply that smoking in pregnancy is safe as robust evidence has been provided in the literature that smoking in pregnancy does cause other negative health outcomes for the fetus.

## Data Availability

ALSPAC data access is through a system of managed open access. The steps below highlight how to apply for access to ALSPAC data: 1) Please read the ALSPAC access policy (https://tinyurl.com/3c623yet) which describes the process of accessing the data and samples in detail, and outlines the costs associated with doing so. 2) You may also find it useful to browse our fully searchable research proposals database (https://proposals.epi.bristol.ac.uk/), which lists all research projects that have been approved since April 2011. 3) Please submit your research proposal for consideration by the ALSPAC Executive Committee. You will receive a response within 10 working days to advise you whether your proposal has been approved. Requests to access these datasets should be directed to alspac-data@bristol.ac.uk.

## References

[1] Lange S Probst C Rehm J Popova S. National, regional, and global prevalence of smoking during pregnancy in the general population: a systematic review and meta-analysis. Lancet Glob Health. (2018) 6:e769–76. doi: 10.1016/S2214-109X(18)30223-7 29859815

[2] Abel EL. Smoking during pregnancy: a review of effects on growth and development of offspring. Hum Biol. (1980) 52:593–625. 7009384

[3] Smith GD. Assessing intrauterine influences on offspring health outcomes: can epidemiological studies yield robust findings? Basic Clin Pharmacol Toxicol. (2008) 102:245–56. doi: 10.1111/j.1742-7843.2007.00191.x 18226080

[4] Castles A Adams EK Melvin CL Kelsch C Boulton ML. Effects of smoking during pregnancy - Five meta-analyses. Am J Prev Med. (1999) 16:208–15. doi: 10.1016/S0749-3797(98)00089-0 10198660

[5] Anderson TM Ferres JML Ren SY Moon RY Goldstein RD Ramirez JM. Maternal smoking before and during pregnancy and the risk of sudden unexpected infant death. Pediatrics. (2019) 143:e20183325. doi: 10.1542/peds.2018-3325 30858347 PMC6564075

[6] World Health Organization. ICD Classifications(2018). Available online at: http://www.who.int/classifications/icd/en/.

[7] Madley-Dowd P Thomas R Boyd A Zammit S Heron J Rai D. Intellectual disability in the children of the Avon Longitudinal Study of Parents and Children (ALSPAC) [version 2; peer review: 1 approved]. Wellcome Open Res. (2023) 7. doi: 10.12688/wellcomeopenres PMC10276197 37333842

[8] Drews CD Murphy CC Yeargin-Allsopp M Decoufle P. The relationship between idiopathic mental retardation and maternal smoking during pregnancy. Pediatrics. (1996) 97:547–53. doi: 10.1542/peds.97.4.547 8632944

[9] Roeleveld N Vingerhoets E Zielhuis GA Gabreels F. Mental-Retardation Associated with Parental Smoking and Alcohol-Consumption before, during, and after Pregnancy. Prev Med. (1992) 21:110–9. doi: 10.1016/0091-7435(92)90010-F 1738762

[10] Mann JR Mcdermott S Barnes TL Hardin J Bao HK Zhou L. Trichomoniasis in pregnancy and mental retardation in children. Ann Epidemiol. (2009) 19:891–9. doi: 10.1016/j.annepidem.2009.08.004 19944351

[11] Mann JR Pan C Rao GA McDermott S Hardin JW. Children born to diabetic mothers may be more likely to have intellectual disability. Maternal Child Health J. (2013) 17:928–32. doi: 10.1007/s10995-012-1072-1 22798077

[12] Huang JC Zhu TT Qu Y Mu DZ. Prenatal, perinatal and neonatal risk factors for intellectual disability: A systemic review and meta-analysis. PloS One. (2016) 11:e0153655. doi: 10.1371/journal.pone.0153655 27110944 PMC4844149

[13] Braun JM Daniels JL Kalkbrenner A Zimmerman J Nicholas JS. The effect of maternal smoking during pregnancy on intellectual disabilities among 8-year-old children. Paediatr Perinatal Epidemiol. (2009) 23:482–91. doi: 10.1111/j.1365-3016.2009.01056.x 19689499

[14] Lundberg F Cnattingius S D'Onofrio B Altman D Lambe M Hultman C. Maternal smoking during pregnancy and intellectual performance in young adult Swedish male offspring. Paediatr Perinatal Epidemiol. (2010) 24:79–87. doi: 10.1111/j.1365-3016.2009.01073.x PMC3653250 20078833

[15] Hirvonen M Ojala R Korhonen P Haataja P Eriksson K Rantanen K. Intellectual disability in children aged less than seven years born moderately and late preterm compared with very preterm and term-born children - a nationwide birth cohort study. J Intellec Disabil Res. (2017) 61:1034–54. doi: 10.1111/jir.12394 28699168

[16] Madley-Dowd P Kalkbrenner AE Heuvelman H Heron J Zammit S Rai D. Maternal smoking during pregnancy and offspring intellectual disability: sibling analysis in an intergenerational Danish cohort. Psychol Med. (2020) 52(10):1847–56. doi: 10.1017/S0033291720003621 PMC8044256 33050963

[17] Madley-Dowd P Lundberg M Heron J Zammit S Ahlqvist VH Magnusson C. Maternal smoking and smokeless tobacco use during pregnancy and offspring development: sibling analysis in an intergenerational Swedish cohort. Int J Epidemiol. (2022) 50:1840–51. doi: 10.1093/ije/dyab095 PMC8743113 34999852

[18] Hammerton G Munafo MR. Causal inference with observational data: the need for triangulation of evidence. Psychol Med. (2021) 51:563–78. doi: 10.1017/S0033291720005127 PMC8020490 33682654

[19] Lawlor DA Tilling K Davey Smith G. Triangulation in aetiological epidemiology. Int J Epidemiol. (2016) 45:1866–86. doi: 10.1093/ije/dyw314 PMC5841843 28108528

[20] Richmond RC Al-Amin A Smith GD Relton CL. Approaches for drawing causal inferences from epidemiological birth cohorts: a review. Early Hum Dev. (2014) 90:769–80. doi: 10.1016/j.earlhumdev.2014.08.023 PMC5154380 25260961

[21] Lipsitch M Tchetgen E Cohen T. Negative controls: a tool for detecting confounding and bias in observational studies. Epidemiology. (2010) 21:383–8. doi: 10.1097/EDE.0b013e3181d61eeb PMC3053408 20335814

[22] Madley-Dowd P Rai D Zammit S Heron J. Simulations and directed acyclic graphs explained why assortative mating biases the prenatal negative control design. J Clin Epidemiol. (2020) 118:9–17. doi: 10.1016/j.jclinepi.2019.10.008 31689456 PMC7001034

[23] Boyd A Golding J Macleod J Lawlor DA Fraser A Henderson J. Cohort Profile: the ‘children of the 90s’–the index offspring of the Avon Longitudinal Study of Parents and Children. Int J Epidemiol. (2013) 42:111–27. doi: 10.1093/ije/dys064 PMC3600618 22507743

[24] Fraser A Macdonald-Wallis C Tilling K Boyd A Golding J Davey Smith G. Cohort Profile: the Avon Longitudinal Study of Parents and Children: ALSPAC mothers cohort. Int J Epidemiol. (2013) 42:97–110. doi: 10.1093/ije/dys066 22507742 PMC3600619

[25] Northstone K Ben Shlomo Y Teyhan A Hill A Groom A Mumme M. The Avon Longitudinal Study of Parents and children ALSPAC G0 Partners: A cohort profile [version 1; peer review: 1 approved with reservations]. Wellcome Open Res. (2023) 8. doi: 10.12688/wellcomeopenres

[26] Chisholm J. The Read clinical classification. BMJ: Br Med J. (1990) 300:1092. doi: 10.1136/bmj.300.6732.1092 2344534 PMC1662793

[27] NHS Digital. Read Codes(2018). Available online at: https://digital.nhs.uk/services/terminology-and-classifications/read-codes.

[28] Department for Education and Skills. Data Collection by Type of Special Educational Need (DfES/0536/2003)(2003). Available online at: http://www.education.gov.uk/publications/eOrderingDownload/DFES-0536–2003.rtf.

[29] R Core Team. R: A language and environment for statistical computing. Vienna, Austria: R Foundation for Statistical Computing (2019).

[30] Rolan-Alvarez E Caballero M. Estimating sexual selection and sexual isolation effects from mating frequencies. Evolution. (2000) 54:30–6. doi: 10.1111/evo.2000.54.issue-1 10937180

[31] Rolan-Alvarez E Carvajal-Rodriguez A de Coo A Cortes B Estevez D Ferreira M. The scale-of-choice effect and how estimates of assortative mating in the wild can be biased due to heterogeneous samples. Evolution. (2015) 69:1845–57. doi: 10.1111/evo.12691 26085130

[32] Hughes RA Heron J Sterne JAC Tilling K. Accounting for missing data in statistical analyses: multiple imputation is not always the answer. Int J Epidemiol. (2019) 48(4):1294–304. doi: 10.1093/ije/dyz032 PMC6693809 30879056

[33] Lee KJ Tilling KM Cornish RP Little RJA Bell ML Goetghebeur E. Framework for the treatment and reporting of missing data in observational studies: The Treatment And Reporting of Missing data in Observational Studies framework. J Clin Epidemiol. (2021) 134:79–88. doi: 10.1016/j.jclinepi.2021.01.008 33539930 PMC8168830

[34] Madley-Dowd P Hughes R Tilling K Heron J. The proportion of missing data should not be used to guide decisions on multiple imputation. J Clin Epidemiol. (2019) 110:63–73. doi: 10.1016/j.jclinepi.2019.02.016 PMC6547017 30878639

[35] van Buuren S. Multiple imputation of discrete and continuous data by fully conditional specification. Stat Methods Med Res. (2007) 16:219–42. doi: 10.1177/0962280206074463 17621469

[36] White IR Royston P Wood AM. Multiple imputation using chained equations: Issues and guidance for practice. Stat Med. (2011) 30:377–99. doi: 10.1002/sim.4067 21225900

[37] van Buuren S Groothuis-Oudshoorn K. Package ‘mice’: Multivariate Imputation by Chained Equations. The Comprehensive R Archive Network: cran.r-project.org (2020).

[38] Rubin DB. Multiple imputation for nonresponse in surveys. New York: Wiley (1987). doi: 10.1002/SERIES1345

[39] Dempster AP Laird NM Rubin DB. Maximum likelihood from incomplete data via the EM algorithm. J R Stat Soc Ser B (Methodol). (1977) 39(1):1–38. doi: 10.1111/j.2517-6161.1977.tb01600.x

[40] Russell T Crawford M Woodby L. Measurements for active cigarette smoke exposure in prevalence and cessation studies: why simply asking pregnant women isn’t enough. Nicotine Tob Res. (2004) 6 Suppl 2:S141–51. doi: 10.1080/14622200410001669141 15203817

[41] Cornish RP Macleod J Boyd A Tilling K. Factors associated with participation over time in the Avon Longitudinal Study of Parents and Children: a study using linked education and primary care data. Int J Epidemiol. (2021) 50:293–302. doi: 10.1093/ije/dyaa192 33057662 PMC7938505

