# Corrigendum: Maternal smoking during pregnancy and offspring risk of intellectual disability: a UK-based cohort study

**DOI:** 10.3389/fpsyt.2024.1520856

**Published:** 2024-11-20

**Authors:** Paul Madley-Dowd, Richard Thomas, Andy Boyd, Stanley Zammit, Jon Heron, Dheeraj Rai

**Affiliations:** ^1^ Centre for Academic Mental Health, Population Health Sciences, Bristol Medical School, University of Bristol, Bristol, United Kingdom; ^2^ National Institute for Health and Care Research (NIHR) Bristol Biomedical Research Centre, University Hospitals Bristol and Weston National Health Service (NHS) Foundation Trust and University of Bristol, Bristol, United Kingdom; ^3^ Medical Research Council Integrative Epidemiology Unit at the University of Bristol, Bristol, United Kingdom; ^4^ UK Longitudinal Linkage Collaboration, Population Health Sciences, Bristol Medical School, University of Bristol, Bristol, United Kingdom; ^5^ Medical Research Council (MRC) Centre for Neuropsychiatric Genetics and Genomics, Cardiff University, Cardiff, United Kingdom; ^6^ Avon and Wiltshire Partnership NHS Mental Health Trust, Bath, United Kingdom

**Keywords:** ALSPAC, intellectual disability, negative control, prenatal exposure, smoking

In the published article, there was an error in the **Funding** statement. Two Wellcome Trust grant numbers were incorrectly given as “092731” and “086118”. This section previously stated:

“The author(s) declare financial support was received for the research, authorship, and/or publication of this article. The UK Medical Research Council and Wellcome (Grant ref: 217065/Z/19/Z) and the University of Bristol provide core support for ALSPAC. A comprehensive list of grants funding is available on the ALSPAC website (http://www.bristol.ac.uk/alspac/external/documents/grant-acknowledgements.pdf); This research was specifically funded by the Wellcome Trust and MRC (Grant refs: 076467/Z/05/Z; 203776/Z/16/A; 092731; 086118; MC_PC_17210). This research was also supported by the National Institute for Health and Care Research Bristol Biomedical Research Centre. PM-D and DR are members of the UK Medical Research Council (MRC) Integrative Epidemiology unit, which is funded by the MRC (MC_UU_00032/02, and MC_UU_00032/6) and the University of Bristol. AB and RT are supported by the UK Longitudinal Linkage Collaboration, which is funded by the MRC and ESRC (MR/X021556/1 and ES/X000567/1) and the University of Bristol. The funders had no role in the design of the study, data management, data analysis, interpretation of findings, and the decision to submit the article for publication.”

The correct statement appears below:

“The author(s) declare financial support was received for the research, authorship, and/or publication of this article. The UK Medical Research Council and Wellcome (Grant ref: 217065/Z/19/Z) and the University of Bristol provide core support for ALSPAC. A comprehensive list of grants funding is available on the ALSPAC website (http://www.bristol.ac.uk/alspac/external/documents/grant-acknowledgements.pdf); This research was specifically funded by the Wellcome Trust and MRC (Grant refs: 076467/Z/05/Z; 203776/Z/16/A; 092731/Z/10/Z; 086118/Z/08/Z; MC_PC_17210). This research was also supported by the National Institute for Health and Care Research Bristol Biomedical Research Centre. PM-D and DR are members of the UK Medical Research Council (MRC) Integrative Epidemiology unit, which is funded by the MRC (MC_UU_00032/02, and MC_UU_00032/6) and the University of Bristol. AB and RT are supported by the UK Longitudinal Linkage Collaboration, which is funded by the MRC and ESRC (MR/X021556/1 and ES/X000567/1) and the University of Bristol. The funders had no role in the design of the study, data management, data analysis, interpretation of findings, and the decision to submit the article for publication.”

The authors apologize for this error and state that this does not change the scientific conclusions of the article in any way. The original article has been updated.

