# Supplementary material for: Maternal smoking during pregnancy and offspring risk of intellectual disability: a UK-based cohort study
Source: Front Psychiatry. 2024 Jun 25;15:1352077. doi: 10.3389/fpsyt.2024.1352077 (PMC11232073; doi:10.3389/fpsyt.2024.1352077)
Supplement: Supplementary file 1 [file Table1.docx]

Supplementary material

Maternal smoking during pregnancy and offspring risk of intellectual disability: a UK based cohort study

Contents

[Supplementary Methods 2](#_Toc152235214)

[Exposure definition 2](#_Toc152235215)

[Covariate definitions 3](#_Toc152235216)

[Details of the multiple imputation procedure and auxiliary variables 4](#_Toc152235217)

[Supplementary Results 5](#_Toc152235218)

[Missing data assessment 5](#_Toc152235219)

[Supplementary Tables 7](#_Toc152235220)

[Supplementary Material References 12](#_Toc152235221)

# Supplementary Methods

## Exposure definition

Maternal smoking is reported at several time points during pregnancy in ALSPAC. Both mothers and partners were asked the following questions in a questionnaire intended to be answered at 18 weeks gestation:

1. Have you ever been a smoker?
2. Have you now stopped smoking?
3. How many times per day did you smoke in the first 3 months of your pregnancy? (For the partner questionnaire this question was worded as “How many times per day did you smoke at the start of your partner’s pregnancy?” but treated equivalently)
4. How many times per day did you smoke in the last 2 weeks?

This time point was selected because it is the only time point at which both maternal and partner smoking were reported, and it uses identical phrasing for all questions but one. The actual timing of completion of the questionnaire varied for both study mothers and partners. The mean (SD) gestation at completion for mothers was 20.8 (5.66) weeks with median (IQR) gestation of 19 (18-21) weeks. For partner responses, the mean (SD) gestation at questionnaire completion was 20.23 (3.50) weeks with median (IQR) gestation of completion equal to 19 [18-21] weeks.

Binary measures of smoking in pregnancy were created for both mother and partner. Smokers were defined as having met Condition 1 or 2 below while a non-smoker was defined as having met neither condition provided data was available for at least one of the conditions:

1. Reported “yes” to Question 1 and had not reported “yes” to question 2 (i.e., reported “no” or left Question 2 missing).
2. The number of cigarettes reported per day in Question 3 or 4 was greater than 0.

A continuous measure of the number of times smoked per day obtained from question 4 (i.e., gestational weeks 16-18). This was captured in categories (0, 1-4, 5-9, 10-14, 15-19,20-24, 25-29, 30+). The lowest number from each category was used to create a conservative estimate of maternal/partner self-report.

ALSPAC also contains maternal report of partner smoking intended for completion at 18 weeks gestation. Mother responses for partner were recorded at the same time as mothers own smoking. Mothers were asked “Does your partner smoke?” and “If yes, about how many times per day does your partner smoke at the moment?” with responses captured in the same categories as for the mother’s own smoking (except with no option for 0 cigarettes smoked at the moment). This may cause issue as the partner may be a smoker but have given up smoking during the pregnancy period. Previous work, however, has shown high concordance between partner’s self-report and maternal report of partner smoking but low concordance for quantity of smoking [1]. Maternal report was therefore used to impute missing partner smoking status (but not quantity) where possible. Here there is a potential trade off in the negative control design between bias from increased error in the paternal report and bias/loss in statistical efficiency from missing data.

## Covariate definitions

Maternal age was grouped as under 25, 25-29, 30-34 and 35 or over. Parity was grouped as 0 (nulliparous), 1, or 2 or greater. Maternal depressive symptoms were measured using the Edinburgh Postnatal Depression Scale. A cut off of 12 was used to create a binary indicator as this has previously been shown to have a high correlation with depression [2]. Maternal alcohol use was included as a binary measure indicating the report of any alcohol use.

Financial difficulties during pregnancy were recorded on a scale from 0-15 with 15 indicating maximum financial difficulty. Mothers were asked “how difficult at the moment do you find it to afford these items?” for each of food, clothing, heating, rent or mortgage and things you will need for the baby. A score greater than 9 was used to indicate that financial difficulties were experienced during pregnancy. This score reflected the 10% of the sample that reported the greatest financial difficulty.

Maternal education was grouped as vocational, CSE/O level and A level/degree. Maternal occupational class was grouped into manual or non-manual occupation according to the definitions provided by the 1991 British Office of Population Census Surveys job codes) [3].

Categorical variables were used in the place of continuous variable for maternal age at birth, maternal depressive symptoms, and financial difficulties. This was done to allow for the possibility of non-linear associations between these variables and the outcome.

The covariates were grouped together for the purpose of investigating different model adjustment strategies. Maternal characteristics included maternal age at birth, parity, maternal depressive symptoms, and maternal alcohol use during pregnancy. Socioeconomic characteristics included financial difficulties, education, and occupational class. Child sex was included with maternal characteristics during adjustments.

Equivalent partner covariates were collected and derived in the same manner as for maternal covariates. Partner education, depression and alcohol use were all reported by the partner at 18 weeks gestation using the same questions/measures as for the mother. Partner occupational class was reported by the mother at the same time as reporting her own occupational class.

## Details of the multiple imputation procedure and auxiliary variables

Binary variables (maternal and partner smoking status during pregnancy, financial difficulties, depression and alcohol use) were imputed using logistic regression, unordered categorical variables (maternal and partner occupational class) were imputed using multinomial logistic regression, ordered categorical variables (parity group and age group) were imputed using proportional odds models, and numeric variables (maternal and partner count exposure variables) were imputed using predictive mean matching.

Homeownership status was grouped as (i) owned/mortgaged, (ii) rented from the council, (iii) privately rented, or (iv) other. Maternal marital status was grouped as (i) never married, (ii) previously married but not currently married (this included those who were widowed, separated, and divorced), (iii) those who were in their first marriage, and (iv) those who were in their second or third marriage. Data on homeownership and marital status were available for 12,572 (93.3%) and 12,628 (93.7%) of the analysis sample respectively.

# Supplementary Results

## Missing data assessment

The distributions of exposure, outcome and covariate variables among participants with no missing data in any variables as compared to participants with data missing in at least one variable are presented in Table S1. Using complete case analysis would lose 34.7% of observations to missing data in any variable for observational analyses. This increased to 61.8% when partner variables were included, as in the negative control analyses.

Table S1, presented separately for the observational analysis and the negative control analysis, can be used to identify associations with the probability of being a missing value and therefore guide understanding of the likely biases that would arise in complete case analyses. Odds ratios for missing data in any variable were produced using unadjusted logistic regression models.

For the observational complete records analyses, having ID was associated with increased odds of having a missing value in any variable. Smokers and heavier smokers were also more likely to be excluded from complete case analyses for missing data. Younger mothers, mothers with greater parity, mothers with depression, and mothers with a manual occupation were also more likely to have missing data. Mothers who used alcohol, mother’s whose ethnicity was white, and mothers who had A levels or a degree were less likely to have missing data in any variable.

For the negative control analyses there were some differences between mothers and partners in the strength of the relationship between variables and being excluded from complete cases analyses for missing data in the negative control analyses. Participants were more likely to be excluded for missing data in any variable if their mother was a smoker than if their mother’s partner was a smoker(maternal OR = 2.10, 95%CI = 1.93-2.28; partner OR = 1.73, 95%CI =1.60-1.86), though the relationship between number of cigarettes smoked per day and missing data was comparable for mother and partner. Missing data in any variable in the negative control analysis was less likely if partners drank alcohol during pregnancy whereas there was no association between maternal alcohol consumption and exclusion from complete records analyses. The association with missing data was similar between mothers and partners for all other covariates.

Table S2 shows the number of observations with missing data in each variable separated by the number of variables missing for the observational analysis. Data are presented for observations with at least one missing value (i.e. only observations excluded from complete records analysis). This table can be used to show the co-occurrence of missing data in multiple variables. Of those excluded from complete records analysis maternal smoking was missing 6.6% of the time and was never missing on its own. No ID information was available for 3.1% of those excluded from complete records analysis and it was frequently missing alongside other variables. Socioeconomic variables were often missing (ranging from 12.6% for maternal education to 31.6% for occupation) and were commonly missing together. Maternal depression, alcohol use and child parity were also frequently missing.

# Supplementary Tables

[Table S1: Descriptive statistics for missing data in any variable for observational and negative control analyses 8](#_Toc151732285)

[Table S2: Counts of missing data in each variable separated by the number of missing variables. 10](#_Toc151732286)

[Table S3: Cross tabulation of maternal and partner smoking during pregnancy 11](#_Toc151732287)

Table S1: Descriptive statistics for missing data in any variable for observational and negative control analyses

|  | Observational analyses | | | Negative control analyses | | |
| --- | --- | --- | --- | --- | --- | --- |
|  | Complete record,  N (%) | Incomplete record, N (%) | OR for being an incomplete record (95% CI) ^a^ | Complete record,  N (%) | Incomplete record, N (%) | OR for being an incomplete record (95% CI) ^a^ |
|  | N = 8808 | N = 4671 |  | N = 5151 | N = 8328 |  |
| Outcome |  |  |  |  |  |  |
| Offspring ID | 81 (0.92) | 56 (1.30) | 1.42 (1.01-2.00) | 34 (0.66) | 103 (1.29) | 1.97 (1.34-2.91) |
|  |  |  |  |  |  |  |
| Exposure |  |  |  |  |  |  |
| Maternal smoking at 18 weeks gestation | 1968 (22.34) | 1433 (36.88) | 2.03 (1.87-2.20) | 958 (18.60) | 2443 (32.39) | 2.10 (1.93-2.28) |
| Maternal smoking quantity among smokers ^b^ | 5 (0-10) | 5 (1-15) | 1.06 (1.05-1.07) | 5 (0-10) | 5 (0-15) | 1.06 (1.05-1.07) |
| Partner smoking at 18 weeks gestation |  |  |  | 1660 (32.23) | 3250 (45.08) | 1.73 (1.60-1.86) |
| Partner smoking quantity among smokers ^b^ |  |  |  | 10 (1-20) | 10 (5-20) | 1.03 (1.03-1.04) |
|  |  |  |  |  |  |  |
| Covariates |  |  |  |  |  |  |
| Child sex |  |  |  |  |  |  |
| Female | 4275 (48.54) | 2256 (48.30) | Ref | 2515 (48.83) | 4016 (48.22) | Ref |
| Male | 4533 (51.46) | 2415 (51.70) | 1.01 (0.94-1.08) | 2636 (51.17) | 4312 (51.78) | 1.02 (0.96-1.10) |
| Parity |  |  |  |  |  |  |
| 0 | 4340 (49.27) | 1268 (34.60) | Ref | 2691 (52.24) | 2917 (39.84) | Ref |
| 1 | 3058 (34.72) | 1284 (35.03) | 1.44 (1.31-1.57) | 1709 (33.18) | 2633 (35.96) | 1.42 (1.31-1.54) |
| >= 2 | 1410 (16.01) | 1113 (30.37) | 2.70 (2.44-2.99) | 751 (14.58) | 1772 (24.20) | 2.18 (1.97-2.41) |
| Maternal age |  |  |  |  |  |  |
| <25 | 1669 (18.95) | 1602 (34.30) | Ref | 875 (16.99) | 2396 (28.77) | Ref |
| 25-29 | 3557 (40.38) | 1647 (35.26) | 0.48 (0.44-0.53) | 2114 (41.04) | 3090 (37.10) | 0.53 (0.49-0.59) |
| 30-34 | 2648 (30.06) | 1018 (21.79) | 0.40 (0.36-0.44) | 1616 (31.37) | 2050 (24.62) | 0.46 (0.42-0.51) |
| >= 35 | 934 (10.60) | 404 (8.65) | 0.45 (0.39-0.52) | 546 (10.60) | 792 (9.51) | 0.53 (0.46-0.61) |
| Paternal age |  |  |  |  |  |  |
| <25 |  |  |  | 417 (8.10) | 387 (15.76) | Ref |
| 25-29 |  |  |  | 1739 (33.76) | 784 (31.92) | 0.49 (0.41-0.57) |
| 30-34 |  |  |  | 1616 (31.37) | 2050 (24.62) | 0.46 (0.42-0.51) |
| >= 35 |  |  |  | 1151 (22.35) | 544 (22.15) | 0.51 (0.43-0.60) |
| Maternal Depression |  |  |  |  |  |  |
| No | 7763 (88.14) | 2331 (80.30) | Ref | 4616 (89.61) | 5478 (83.51) | Ref |
| Yes | 1045 (11.86) | 572 (19.70) | 1.82 (1.63-2.04) | 535 (10.39) | 1082 (16.49) | 1.70 (1.53-1.90) |
| Partner Depression |  |  |  |  |  |  |
| No |  |  |  | 4994 (96.95) | 4138 (94.84) | Ref |
| Yes |  |  |  | 157 (3.05) | 225 (5.16) | 1.73 (1.40-2.13) |
| Maternal Alcohol Use |  |  |  |  |  |  |
| No | 3915 (44.45) | 1778 (47.53) | Ref | 2333 (45.29) | 3360 (45.42) | Ref |
| Yes | 4893 (55.55) | 1963 (52.47) | 0.88 (0.82-0.95) | 2818 (54.71) | 4038 (54.58) | 0.99 (0.93-1.07) |
| Partner Alcohol Use |  |  |  |  |  |  |
| No |  |  |  | 177 (3.44) | 279 (6.63) | Ref |
| Yes |  |  |  | 4974 (96.56) | 3932 (93.37) | 0.50 (0.41-0.61) |
| Maternal Ethnicity |  |  |  |  |  |  |
| All other ethnic groups combined | 179 (2.04) | 131 (4.19) | Ref | 80 (1.56) | 230 (3.40) | Ref |
| White | 8585 (97.96) | 2996 (95.81) | 0.48 (0.38-0.60) | 5055 (98.44) | 6526 (96.60) | 0.45 (0.35-0.58) |
| Partner Ethnicity |  |  |  |  |  |  |
| All other ethnic groups combined |  |  |  | 88 (1.72) | 184 (4.31) | Ref |
| White |  |  |  | 5038 (98.28) | 4081 (95.69) | 0.39 (0.30-0.50) |
| Maternal Occupation |  |  |  |  |  |  |
| Non-Manual | 7089 (80.48) | 682 (76.03) | Ref | 4261 (82.72) | 3510 (77.08) | Ref |
| Manual | 1719 (19.52) | 215 (23.97) | 1.30 (1.11-1.53) | 890 (17.28) | 1044 (22.92) | 1.42 (1.29-1.57) |
| Partner Occupation |  |  |  |  |  |  |
| Non-Manual |  |  |  | 3256 (63.21) | 2635 (48.75) | Ref |
| Manual |  |  |  | 1895 (36.79) | 2770 (51.25) | 1.81 (1.67-1.95) |
| Maternal Education |  |  |  |  |  |  |
| Vocational | 835 (9.48) | 350 (11.04) | Ref | 468 (9.09) | 717 (10.50) | Ref |
| CSE/ O level | 4472 (50.77) | 2085 (65.77) | 1.11 (0.97-1.27) | 2450 (47.56) | 4107 (60.16) | 1.09 (0.96-1.24) |
| A level/Degree | 3501 (39.75) | 735 (23.19) | 0.50 (0.43-0.58) | 2233 (43.35) | 2003 (29.34) | 0.59 (0.51-0.67) |
| Partner Education |  |  |  |  |  |  |
| Vocational |  |  |  | 364 (7.07) | 418 (9.74) | Ref |
| CSE/ O level |  |  |  | 2029 (39.39) | 2171 (50.58) | 0.93 (0.80-1.09) |
| A level/Degree |  |  |  | 2758 (53.54) | 1703 (39.68) | 0.54 (0.46-0.63) |
| Financial Difficulties |  |  |  |  |  |  |
| No | 8086 (91.80) | 2403 (84.35) | Ref | 4818 (93.54) | 5671 (87.17) | Ref |
| Yes | 722 (8.20) | 446 (15.65) | 2.08 (1.83-2.36) | 333 (6.46) | 835 (12.83) | 2.13 (1.87-2.43) |

^a^ ORs for being a missing value across levels of the variable
^b^ Values presented for this row are median (IQR) and OR represents the change in odds of being a missing value for a 1 cigarette per day increase among smokers only.

Table S2: Counts of missing data in each variable separated by the number of missing variables.

|  | Number of missing variables, N(%) ^a^ | | | | | | | |  |
| --- | --- | --- | --- | --- | --- | --- | --- | --- | --- |
| Missing variable | 1 | 2 | 3 | 4 | 5 | 6 | 7 | 8 | Total |
| Total | 2335 | 339 | 894 | 403 | 134 | 66 | 414 | 86 | 4671 |
| ID | 57 (2.44) | 50 (14.75) | 12 (1.34) | 120 (29.78) | 31 (23.13) | 15 (22.73) | 2 (0.48) | 86 (100.00) | 373 (3.12) |
| Maternal smoking | 0 (0.00) | 0 (0.00) | 0 (0.00) | 134 (33.25) | 96 (71.64) | 55 (83.33) | 414 (100.00) | 86 (100.00) | 785 (6.56) |
| Child sex | 0 (0.00) | 0 (0.00) | 0 (0.00) | 0 (0.00) | 0 (0.00) | 0 (0.00) | 0 (0.00) | 0 (0.00) | 0 (0.00) |
| Parity ^b^ | 128 (5.48) | 58 (17.11) | 4 (0.45) | 156 (38.71) | 105 (78.36) | 55 (83.33) | 414 (100.00) | 86 (100.00) | 1006 (8.41) |
| Maternal age | 0 (0.00) | 0 (0.00) | 0 (0.00) | 0 (0.00) | 0 (0.00) | 0 (0.00) | 0 (0.00) | 0 (0.00) | 0 (0.00) |
| Maternal depression | 442 (18.93) | 133 (39.23) | 249 (27.85) | 258 (64.02) | 120 (89.55) | 66 (100.00) | 414 (100.00) | 86 (100.00) | 1768 (14.78) |
| Maternal alcohol use | 41 (1.76) | 34 (10.03) | 15 (1.68) | 152 (37.72) | 122 (91.04) | 66 (100.00) | 414 (100.00) | 86 (100.00) | 930 (7.78) |
| Maternal occupation | 1618 (69.29) | 311 (91.74) | 892 (99.78) | 269 (66.75) | 118 (88.06) | 66 (100.00) | 414 (100.00) | 86 (100.00) | 3774 (31.56) |
| Maternal education | 23 (0.99) | 35 (10.32) | 637 (71.25) | 255 (63.28) | 40 (29.85) | 13 (19.70) | 412 (99.52) | 86 (100.00) | 1501 (12.55) |
| Financial difficulties | 26 (1.11) | 57 (16.81) | 873 (97.65) | 268 (66.50) | 38 (28.36) | 60 (90.91) | 414 (100.00) | 86 (100.00) | 1822 (15.24) |

^a^ Percentage for each column sums to 100 × the number of missing variables.
^b^ Example table interpretation: 2335 individuals have data missing in 1 variable only, 128 of those have data missing in the parity variable. 339 individuals have data missing in 2 variables, for 58 individuals, one of those variables is parity.

Table S3: Cross tabulation of maternal and partner smoking during pregnancy

|  |  | Maternal smoking status, N (%) | |
| --- | --- | --- | --- |
|  |  | Non-smoker | Smoker |
| Partner smoking status | Non-smoker | 6481 (71.02) | 955 (29.78) |
|  | Smoker | 2644 (28.98) | 2252 (70.22) |

IPSI value = 0.41

# Supplementary Material References

1. Passaro, K.T., et al., *Agreement between self and partner reports of paternal drinking and smoking. The ALSPAC Study Team. Avon Longitudinal Study of Pregnancy and Childhood.* Int J Epidemiol, 1997. **26**(2): p. 315-20.

2. Cox, J.L., et al., *Validation of the Edinburgh Postnatal Depression Scale (EPDS) in non-postnatal women.* J Affect Disord, 1996. **39**(3): p. 185-9.

3. Office of population censuses and surveys and General register office for Scotland, *1991 Census: Definitions Great Britain*. 1992, HMSO: London.
